# Response to “Sequential Portal Vein–Hepatic Vein Embolization: Progress Yet Unaccounted Pitfalls”

**DOI:** 10.1002/ags3.70175

**Published:** 2026-01-13

**Authors:** Thanh Tung Lai, Masaki Kaibori

**Affiliations:** ^1^ Department of Hepatobiliary Surgery Kansai Medical University Osaka Japan; ^2^ Department of Surgery Hanoi Medical University Hanoi Vietnam

Dear Editor,

We sincerely thank the authors for their thoughtful and constructive comments [1] on our study, which investigated the efficacy of sequential PVE‐HVE prior to right‐sided major hepatectomy [2]. We appreciate the opportunity to clarify several points.

First, regarding preoperative liver function assessment, current global practice primarily relies on future liver remnant volumetry combined with standardized clinical scoring systems, such as Child–Pugh and MELD scores, together with indocyanine green–based tests (ICG‐R15 and ICG‐Krem). Although advanced functional imaging modalities—including 99mTc‐GSA scintigraphy, 99mTc‐mebrofenin hepatobiliary scintigraphy, and EOB‐MRI—provide valuable information, they are not routinely used in many centers because of limited availability, regulatory restrictions, and cost. Consequently, the choice of functional assessment depends on institutional resources and is not mandatory before hepatectomy. In our study, 99mTc‐GSA scintigraphy was used to provide complementary functional information, as GSA binds directly to the asialoglycoprotein receptor on hepatocyte membranes and, unlike EOB‐MRI or mebrofenin scintigraphy, is independent of biliary excretion and less affected by hyperbilirubinemia. The GSA‐Rmax cutoff was defined in a previous study [3] and represents only one component of the embolization indication. Importantly, the primary aim of our study was to evaluate changes in liver function before and after embolization rather than to establish a universal cutoff value.

Second, we acknowledge the limitation of lacking long‐term oncological endpoints. However, the primary objective of this study was to determine whether sequential PVE–HVE could increase resectability. Conversion from unresectable to resectable disease is generally associated with improved prognosis. Evaluation of survival or recurrence would require large, homogeneous cohorts focusing on a single disease entity, which is challenging given the limited indications for this technique and represents a common limitation in previously published PVE–HVE studies.

Third, regarding tumor progression, among the 47 patients who underwent embolization, three did not proceed to hepatectomy: one declined surgery despite meeting resection criteria, one patient in the PVE group, and one in the PVE–HVE group experienced tumor progression. Given the small number of progression events and our focus on surgical outcomes, the two patients with post‐embolization progression were excluded from the surgical outcome analysis.

Fourth, the goal of HVE is to block venous outflow from the right liver. As the presence and size of the IRHV vary among patients, embolization was performed only when it was considered hemodynamically significant, reflecting individualized, anatomy‐based decision‐making.

Eleven center design and the absence of cost‐effectiveness analysis. However, patient safety was our highest priority. While simultaneous embolization may reduce anesthesia time, it may increase risk, particularly in centers newly adopting this technique and in patients with compromised liver parenchyma. Therefore, we adopted a sequential approach to carefully assess liver injury after PVE before proceeding to HVE. Moreover, sequential PVE–HVE aims to accelerate liver hypertrophy, shortening the waiting period to approximately 3–4 weeks and potentially reducing the risk of tumor progression. A 6–8 week assessment is not necessary.

In conclusion, we appreciate the insightful comments and agree that future multicenter, prospective studies with standardized protocols are essential to address current limitations, as also highlighted in our discussion and future research plans.

## Author Contributions

**Thanh Tung Lai:** conceptualization, writing – original draft, writing – review and editing. **Masaki Kaibori:** conceptualization, supervision, writing – review and editing, project administration. All authors approved the final version and agree to be accountable for all aspects of the work, ensuring the accuracy and integrity of the data and interpretation.

## Funding

The authors have nothing to report.

## Conflicts of Interest

The authors declare no conflicts of interest.

## Linked Article

This article is linked with Shahid et al. *Annals of Gastroenterological Surgery* 2025. https://doi.org/10.1002/ags3.70148.
